# Comparing Environmental Policies to Reduce Pharmaceutical Pollution and Address Disparities

**DOI:** 10.3390/ijerph19148292

**Published:** 2022-07-07

**Authors:** Meghana Desai, Anuli Njoku, Lillian Nimo-Sefah

**Affiliations:** 1 Health Analytics Network LLC, Pittsburgh, PA 15237, USA; 2 Department of Public Health, College of Health and Human Services, Southern Connecticut State University, New Haven, CT 06515, USA; njokua3@southernct.edu (A.N.); nimosefahl1@southernct.edu (L.N.-S.)

**Keywords:** pharmaceutical waste, pharmaceutical pollution, environmental policies, disparities

## Abstract

Pharmaceutical products, including active pharmaceutical ingredients and inactive ingredients such as packaging materials, have raised significant concerns due to their persistent input and potential threats to human and environmental health. Discourse on reducing pharmaceutical waste and subsequent pollution is often limited, as information about the toxicity of pharmaceuticals in humans is yet to be fully established. Nevertheless, there is growing awareness about ecotoxicity, and efforts to curb pharmaceutical pollution in the European Union (EU), United States (US), and Canada have emerged along with waste disposal and treatment procedures, as well as growing concerns about impacts on human and animal health, such as through antimicrobial resistance. Yet, the outcomes of such endeavors are often disparate and involve multiple agencies, organizations, and departments with little evidence of cooperation, collaboration, or oversight. Environmental health disparities occur when communities exposed to a combination of poor environmental quality and social inequities experience more sickness and disease than wealthier, less polluted communities. In this paper, we discuss pharmaceutical environmental pollution in the context of health disparities and examine policies across the US, EU, and Canada in minimizing environmental pollution.

## 1. Healthcare Waste and Environmental Pollution

Healthcare waste continues to persist in the environment, leading to pollution and contamination, thereby threatening the integrity of the ecosystem and human health. The World Health Organization (WHO) defines healthcare waste as “all waste related to medical procedures, including waste generated within healthcare facilities, laboratories, research centers, home healthcare, and other minor and scattered sources” [1].The United States (US) alone generates an estimated 5–6 million tons (4.5–5.4 metric tons) of waste each year, often disposed of through incineration, landfilling, and chemical and thermal disinfection [2]. The WHO estimates that approximately 85% of healthcare waste is non-hazardous, and 15% is infectious, toxic, or radioactive [3,4]. In 2020, the leading company in the industry, Waste Management, processed and incinerated over 23 million pounds (~10 million kilograms) of infectious waste [5]. Although healthcare waste represents a relatively smaller proportion of the total waste generated in the community, it is nevertheless considered an important issue around the globe due to its potential for environmental pollution and impact on human, animal, and plant health.

The COVID-19 pandemic has altered waste streams globally and in the first 10 months of the pandemic, researchers reported increased waste globally, ranging from 18–425% depending on the nation and analysis [6]. Every month, 129 billion face masks and 65 billion gloves are used to protect citizens worldwide and few healthcare facilities rely on reusable types of respiratory protection [7]. Healthcare waste consists of several types of waste, including medical waste plastics, and other plastic items [7,8,9,10]. Each day, 20–25% of healthcare waste can be attributed to plastic packaging and products [11]. In the US, during the pandemic, several recycling programs were paused due to concerns about the risk of contamination, leading to an increase in incineration and landfilling to manage medical waste [7], thereby leading to concerns regarding potential toxic and hazardous air emissions along with ground and surface water pollution [12,13]. This poses a significant problem for rapid disposal and ensuring environmental safety and justice. 

Pharmaceuticals were identified to pose environmental risks in the 1990s, and active pharmaceutical ingredients (APIs) are found in sewage water, surface water, groundwater, soil, air, or biota in concentrations from sub-ng/L to more than μg/L [14,15]. Among the different categories of healthcare waste, pharmaceutical waste can have harmful effects on the environment, even in small concentrations, such as renal failure in vultures, impairment of reproduction in fish, or inhibition of certain aquatic species [16,17,18]. The discharge of pharmaceuticals into the environment has been linked to the development of antimicrobial resistance, which is recognized as one of the greatest public health challenges in the 21st century [19]. Acute and chronic exposures to pharmaceuticals in aquatic systems can disrupt ecological processes. For example, selective serotonin reuptake inhibitors (SSRIs) such as Prozac, and Celexa, are prescribed as antidepressants and are commonly detected in surface waters and can lead to altered growth, reproduction, and behavior (aggression, boldness) in aquatic invertebrates [18,19,20,21,22]. See Table 1 for types of pharmaceutical waste.

In the context of environmental pollution, a broader category known as pharmaceutical and personal care products (PPCPs) includes prescription and non-prescription drugs, diagnostic agents, nutraceuticals, sunscreens, and fragrances, among other products. Pharmaceuticals that are designed to be slowly degradable or even nondegradable to resist chemical degradation during passage through a human or animal body present a special risk when they enter, persist, or disseminate in the environment. Such substances are referred to as environmentally persistent pharmaceutical pollutants (EPPPs) [23] 

Pathways for pollution: Pharmaceuticals are released into the environment from wastewater treatment systems, aquaculture facilities, runoffs from fields, and releases to solids during biosolid and manure applications [24].

Broadly, pharmaceuticals are divided into two categories based on their consumption—veterinary and human. Veterinary pharmaceuticals are used for aquaculture, companion animals, and livestock and find their way into the environment by way of their disposal. In particular, livestock pharmaceuticals are often found in manure or biowaste that eventually find their way to freshwater and terrestrial ecosystems. Human pharmaceuticals are used by individuals in their own households or in healthcare facilities from where they make their way into wastewater and/or solid waste disposal, and eventually into freshwater and terrestrial ecosystems. Environmental reservoirs of antibiotic-resistant bacteria are driven by the indiscriminate disposal of antibiotics and antimicrobials into the ecosystem, thereby driving drug resistance. 

Pharmaceuticals are ideally disposed of by incineration at high temperatures (above 1200 °C) with adequate flue gas cleaning. However, this has not been the case globally and growing concerns over PPCP and EPPP presence in the environment have led to discussions on policies and guidelines on appropriate waste disposal and management. However, as pharmaceutical waste is produced, handled, and processed, policy and programmatic protections are much needed to address environmental health and justice.

## 2. Social Determinants of Health and Environmental Health Disparities

The social determinants of health are non-medical factors that influence health outcomes and include individual behavior, biology, socioeconomic status, physical and social environment, discrimination, racism, access to affordable health services, and legislative policies [25,26]. The social determinants of health are largely responsible for health inequities, or unfair and avoidable differences in health status between countries and between different groups of people within the same country [27,28]. Addressing the underlying determinants of health to reduce health inequities is imperative because health is a fundamental human right, and the failure to overcome inequities results in health disparities [29,30]. Environmental conditions play a key role in producing and maintaining health disparities [31].

Environmental health disparities occur when communities exposed to a combination of poor environmental quality and social inequities experience more sickness and disease than wealthier, less polluted communities [32]. Environmental equity can indicate an equal sharing of environmental risk burdens and the effects of environmental degradation [33]. Environmental justice signifies remedial action to correct an injustice imposed on a specific group of people, mostly people of color in the US [34]. It can be accomplished through the development, implementation, and enforcement of environmental laws, regulations, and policies to ensure everyone receives equal protection from environmental and health hazards and the same access to the decision-making process for a healthy environment [35]. Moreover, there is a need to carefully examine environmental health disparities and how systems and policies create and perpetuate inequalities in exposure to environmental pollutants among communities of color and vulnerable populations, putting them at increased risk for disease and mortality [36,37].

The growing creation of healthcare waste contributes to the increasing incidence of chronic disease [38]. Throughout the world, people that suffer the most from the pharmaceutical pollution crisis often contribute the least to the problem. The practices of inequitable regulation, generation, storage, treatment, and disposal of pharmaceutical pollutants is an environmental justice issue that also encourages the exploration of the social determinants of health [39]. While research on toxic exposures in vulnerable populations often focuses on air pollution, synthetic chemicals released into the environment as pollutants from waste disposal can contribute to global health disparities [40]. 

Indigenous peoples and communities in North America include groups of Aboriginal peoples such as the Indians (often referred to as First Nations), Inuit, and Métis in Canada [41]. An assessment of pharmaceuticals in source waters on the reserve lands of 95 First Nations in Canada revealed that quantifiable levels of 35 pharmaceuticals were found in approximately 83% of participating First Nations at 68% of surface water sites [42]. 

Results concluded that surface water in the surrounding area of major urban centers should not be used as supplementary untreated water sources due to increased human and ecological health risks, related to exposure to mixtures of several pharmaceuticals that have been detected [42,43]. Moreover, the U.S. government formally recognizes 574 Indian tribes in the contiguous 48 states and Alaska [44]. In the U.S., there has been a destructive assault on Indian (Tribal) lands as countless Indian Nations (Tribes) have been approached by the US government and waste disposal industry in search of new dumping grounds for the unwanted medical, solid, toxic, and nuclear waste (e.g., pesticides, asbestos, sewage sludge ash) of industrial society [45,46]. Indian Nations have resisted the toxic invasion of the waste industry by rejecting proposal agreements for medical waste incinerators outright [45]. Impoverished rural U.S. populations also experience health disparities due to inequitable exposures to environmental toxins [47]. Rural Appalachia, a cultural region in the US that stretches from southern New York to northern Mississippi, has experienced health disparities and environmental justice concerns from the siting of medical incinerators that have produced uncontrollable emissions [48].

Furthermore, most developing countries are unable to successfully manage their medical waste because of a shortage of resources, ineffective management of available resources, and limited transparency in administration [49,50]. In South Africa, incineration is the most frequently used method to dispose of toxic medical waste; still, incinerators are known to pollute the air, soil, and surface water by emitting toxic chemicals into the atmosphere, and incinerator use has been associated with the disruption of human immune, hormonal, and reproductive systems, and cancers [51,52]. Countries such as Bangladesh have experienced an environmental catastrophe of personal protective equipment (PPE) disposal and management during COVID-19, risking biodiversity and contributing to irreversible damage to ecosystems [53]. A study in Sudan revealed that the management of healthcare and home-generated healthcare waste, particularly used needles, in the country of about 40 million people is inefficient and places waste workers and the environment at risk. Overall, healthcare waste disparities represent a major public health issue, particularly in disadvantaged communities worldwide that often bear the greatest burdens of environmental degradation. 

High-income countries in the Northern hemisphere have policies and regulations to address and mitigate risks from environmental pollution, especially around toxic waste, whereas middle and low-income countries often lack the necessary environmental protections and practices to prevent and mitigate risks from pharmaceutical dumping. For example, the WHO lists antibiotic resistance (AMR) as one of the greatest threats to human health. While AMR can occur naturally, the misuse of antibiotics in humans and animals exacerbates this problem. In many parts of the world, antibiotics are often available for purchase over-the-counter and are disposed of improperly. 

Antimicrobial resistance policies: In 2013, the US Food and Drug Administration (FDA) finalized Guidance #213, a policy to limit antibiotics for production purposes (i.e., given to healthy animals to promote growth and enhance feed efficiency). Guidance #213 was fully implemented in January 2017 and the addition of antibiotics to feed and water requires the oversight of a veterinarian. A companion policy, the Veterinary Feed Directive, issued in 2015, outlined the roles and responsibilities of supervising veterinarians. Further, in 2019, the FDA issued Guidance #263 for the industry to voluntarily change the marketing status of certain over-the-counter antimicrobials to prescription only that would necessitate a veterinarian’s supervision [54,55]. The Canadian government has a multi-department federal action framework to limit antimicrobial resistance through collaborations, surveillance, and stewardship [56]. The EU adopted a One Action Health Plan against AMR in 2017 [57]. The problem of AMR has been thus far handled from the viewpoint of health rather than environmental contamination. While results of the AMR stewardship programs and policies remain to be seen in terms of reducing antimicrobials in the environment, inclusion or collaboration with environmental groups and agencies may further enable a cycle of pollution prevention through reduced usage, active surveillance and monitoring, and rapid responses to environmental contamination.

## 3. Pharmaceutical Pollution Policies

The information on pharmaceutical pollution policies and disparities was collected through literature searches, policy searches, and a review of policy documents. In this paper, policy statements from the US, EU, and Canada were reviewed for guidance, regulations, and enforcement regarding pharmaceutical waste and pollution. A simple checklist was used to identify current policies pertaining to pharmaceutical pollution.

## 4. Policies Addressing Pharmaceutical Pollution

### 4.1. Policies for Pharmaceutical Disposal and Preventing Environmental Pollution Are Often Developed and Adopted by Different Countries

Most industrialized nations have developed policies to minimize environmental impact, however, there is uneven enforcement, regulation, and monitoring to mitigate environmental risks. In the US, multiple agencies such as the Environmental Protection Agency (EPA), National Oceanic and Atmospheric Administration (NOAA), Food and Drug Administration (FDA), and others address environmental pollution in their own sectors. Pharmaceutical waste is regulated by the EPA, Drug Enforcement Agency (DEA), Department of Transportation (DOT), Fish and Wildlife Services (FWS), Occupational Safety and Health Administration (OSHA), and The Joint Commission (JC). The European Commission (EC) has developed policies for environmental pollution in the EU, and in Canada, the Council of Ministers of the Environment develops policies pertaining to environmental waste.

**United States (US):** In the US, pharmaceuticals are considered as chemicals first and then therapeutic agents by the EPA. The EPA and the DEA recommend the incineration of medical waste [58]. However, there are *no official, uniform guidelines* on managing PPCPs in the nation. Each state has the authority to choose how they dispose of waste. Often, pharmaceuticals in households are disposed of in the trash or in sewage. It has been thought that PPCP pollution may be reduced through their proper disposal [50]. However, it may be important to consider the broader usage of pharmaceuticals and limiting their use through stewardship programs along with enforcement of manufacture–usage–disposal practices that limit waste and pollution.

While all types of PPCPs have not been considered yet, there is minor progress in their management. In 2008, the EPA proposed to add pharmaceuticals to the kinds of hazardous wastes that could be managed as Universal Wastes. The EPA currently has a rule geared toward one type of PPCP, nicotine, titled “Management Standards for Hazardous Waste Pharmaceuticals and Amendment to the P075 Listing for Nicotine,” signed in 2018, and published in 2019. The rule supports the nation’s move toward returning unused PPCPs [59].

**Canada**

Several jurisdictions in Canada do not have official policies or regulations governing PPCPs. There is less regulation for pharmaceutical and medical waste when compared to the US. Similar to the US, existing guidelines and recommendations for waste disposal are enforced at the discretion of each province or municipality [60]. The country’s goal is to reach the minimum national standards for managing medical waste set by the Canadian Council of Ministers of the Environment in 1992 [61]. To minimize pharmaceutical waste, Canada also partakes in drug take-back programs for people to return unused pharmaceutical products. The Guidelines for the Management of Biomedical Waste in the Northwest Territories (2005) specify that pharmaceutical products must be segregated from general waste and handled by incineration or chemical neutralization [62]. This is still widely followed and serves as the major guideline for pharmaceutical waste in the country. However, the impact of these programs on reducing environmental pollution *remains unclear*.

**European Union**

In 2013, the EC resolved to minimize water pollution from pharmaceutical products. In response, a 12-week consultation period was conducted to develop an approach for limiting pharmaceutical waste in the environment [63]. The deadline for implementing this approach was in 2018. However, there is little to no evidence of a follow-up on the matter.

The EU subsequently developed a document named “The European Green Deal”. This follows the EC’s attempt to manage environmental pollution. The Green Deal aimed to adopt a “zero pollution action plan” by 2021 [64]. Notably, within this document, the discussion of pharmaceutical products appeared only once. An example of the recommendations to be included in the new approach include the European Medicines Agency working along with the Commission to reduce pharmaceutical waste. This includes developing policies to reduce the packaging size of pharmaceuticals that would amount to reductions in disposal [64].

Similar to the US and Canada, the EU also maintains that incineration is the best method for medical waste disposal. In general, the EU has made a concerted effort to create uniform policies and regulations to be followed by all countries in the Union and to disallow individual countries to decide on how to manage specific types of waste.

Table 2 provides an overview of policies and guidelines on regulating the production, management, and disposal of pharmaceutical products across the US, Canada and EU.

Table 3 below depicts a simple checklist to identify whether policies pertaining to pharmaceutical pollution are compliant, have been enforced, and have yielded results. In all three regions, policies and guidelines exist but there is little evidence of improved outcomes and more importantly of stakeholder/customer consultation. It may be worth considering the development of tools and measures to evaluate whether any of the policies are (a) implementable and/or (b) enforceable through regulations. For instance, including consumer/patient perspectives or participation may likely enable agencies to increase their social commitments towards recycling and reusing, allowing for a better implementation of efforts aimed at reducing pharmaceutical pollution. Similarly, including a diverse set of stakeholders may enable buy-ins for different legislative actions.

### 4.2. Policy Impact and Assessment: Policies Are Only as Good as Their Systematic Implementation and Evaluation

The literature indicates that across the US, Canada and EU, broader efforts are being directed at managing pharmaceutical waste through either upgrading wastewater management systems or drug take-back programs. While it is challenging to adequately measure the impacts of various policies across different time points and geographies, policy implementation and challenges for the three regions are discussed below.

U.S.: The EPA established a series of guidelines in 1985 to derive ambient water quality criteria for aquatic life. However, nationwide standards or guidelines to minimize pharmaceuticals in the environment do not exist. Some regulatory mandates relevant to PPCPs include: the Safe Water Drinking Act (1974 and 1996), Clean Water Act (1972), Resource Conservation and Recovery Act (1976), and the Federal Insecticide, Fungicide, and Rodenticide Act (1948). The EPA’s National Exposure Research Laboratory (NERL) has developed new chemical analytic approaches for PPCPs. It should be noted that the US is one of two countries in the world (the other one is New Zealand) that allows direct-to-consumer advertising of pharmaceuticals thereby driving market demand.

Canada: Information generated through environmental impact assessments and the Canadian Integrated Program for Antimicrobial Resistance (CIPARs) is often publicly unavailable or not made available in a timely and comprehensive manner. Federal wastewater regulations lack specific requirements for managing pharmaceutical pollutants. Similar to the US, there is a lack of consistent information from governmental sources on the safe disposal of unused and expired pharmaceuticals. Several initiatives addressing APIs, such as the PPCP Surveillance Network remain as mostly disconnected efforts, rather than being a strategic vision for systematic surveillance and monitoring [65]. A 2014 report by the Federal Standing Senate Committee on Social Affairs, Science and Technology found that the PPCP Surveillance Network’s activities did not constitute a systematic sampling and reporting program. Rather, it was an informal effort by scientists who were involved in other surveillance and research programs. Likewise, the Committee found that access to the CIPARS data is limited, is not available in a timely and comprehensive manner, and has not been used to its full potential [65].

EU: In 2006, the European Medicines Agency’s (EMA) guideline on environmental risk assessment was implemented in accordance with Article 8(3)—the directive for medicinal products for human use. Briefly, the predicted environmental concentration (PEC) for surface water is calculated and the octanol–water partition coefficient (Kow) is measured. If the PEC value is equal to or above 0.01 μg/L, a Phase II assessment is performed. APIs with a logKow > 4.5 are screened for PBT properties (Persistence, Bioaccumulation, Toxicity) [66]. Per the EMA guideline, ERAs should be performed by companies and evaluated by regulators. Additionally, amendments to Directive 2004/27/EC Article 127b require EU member states to establish ‘an appropriate collection system’ for unused and/or expired medication. However, there has been a lack of follow-up and publicly available information to evaluate this policy.

## 5. Global Policy Framework

Currently, frameworks and guidelines to address pharmaceutical and personal product waste exist to differing degrees across the US, Canada, and Europe. In this paper, we focused on Western regions that have the highest contribution of greenhouse gas emissions and the ability to develop and implement policies enforcing the reduction of waste. Among these, the EU appears to have a higher commitment and specific plans for implementation to reduce environmental pollution by pharmaceuticals through the philosophy of a circular economy.

Circular economy (CE): is a holistic philosophy in waste management for managing and preserving of resources that are currently in use for as long as possible through recovery and reuse. The European Commission adopted a CE approach in 2020. In the US, the EPA provides guidance for CE in reducing environmental waste.

**Figure 1:**
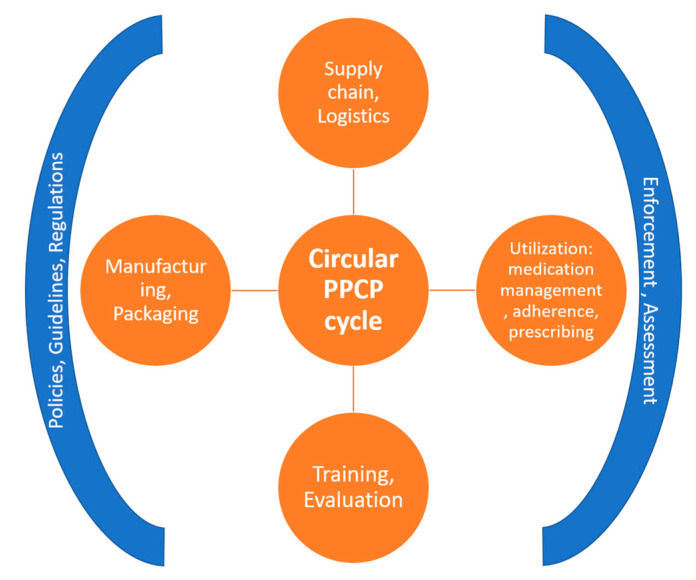
Circular economy applied to PPCPs.

PPCPs are a special category of waste, and the CE philosophy, if applied correctly, has the potential to minimize the presence of pharmaceutical pollutants in the environment. However, this would require disparate groups, sectors, policymakers, and regulatory agencies to come together to develop a binding covenant of measures that would be necessary for a strategic plan to create a continuous loop of use and reuse. Systemic changes to identify points of disposal and recycling, sorting at the source, reducing the amount of disposable containers, identifying practices for better medication management, training healthcare workers, patients, and the general public, and evaluating and improving supply chains are some of the strategies that could lead to waste minimization [67] (see Figure 1). Needless to say, these approaches would require multisectoral commitment and cooperation to be successful.

A core principle of a circular economy is that the value of products and the materials they are made of can be preserved by keeping them in the economic system, either by lengthening the life of the products formed from them or by “looping” them back into the system to be reused [68]. The global healthcare sector is growing rapidly due to aging populations. During COVID-19, single-use and disposable personal protective equipment contributed to solid waste. Yet, the implementation of CE in healthcare and specifically in PPCPs is challenging. There is an inherent contradiction since, in the medical field, the use of disposable syringes, gloves, etc. has led to a reduction in infections and, subsequently, an improvement in human health and mortality. Therefore, it may not be possible to entirely eliminate single-use or disposable items in healthcare, nor pharmaceuticals, however, solutions to improve material deterioration and reduce their persistence in the environment are under consideration by various regulators.

In the pharmaceuticals sector, the entire lab-to-waste process needs to be carefully tracked and evaluated for environmental impact. The Pharmaceuticals Strategy for Europe proposes to ensure greater transparency across the global manufacturing chain through bilateral and multilateral agreements. Tracing the origin of active pharmaceutical ingredients (APIs) in medicinal products is almost impossible due to a globalized supply chain, and such a lack of transparency does not allow for scrutiny of the environmental and human rights risks involved in the pharmaceutical industry’s manufacturing processes. It should be noted here that the EU strategy further proposes to strengthen environmental risk assessments (ERAs). This is an area of regulatory concern since there are no penalties for non-compliance. As such, pharmaceutical manufacturers do not prioritize ERAs in their marketing authorization applications. For example, between 2011–2012, 37% of ERAs were submitted late and 83% were missing or of an unsatisfactory quality [69].

The literature in the healthcare domain has remained focused on infection prevention or cost reduction as compared to environmental pollution. Historically, the terms ecology and human health have been used independently, and only in recent years with the emergence of AMR has there been increasing awareness of the complex dynamic between environmental and human health.

AMR stewardship programs aim to reduce antibiotic misuse. Yet, the impact of such programs on environmental pollution has not been assessed. This presents both an opportunity and a challenge—to educate and train healthcare personnel, and strategically design and implement processes to minimize environmental pollution. Applying the CE framework would require changes in the entire lifecycle of PPCPs—reductions in synthesis and manufacturing to usage, waste disposal, and management to encompass recycling, reuse, and reduction of waste generation (the three Rs of CE).

Another important consideration for the CE framework is an increasing emphasis on “green chemistry”—designing chemicals that would reduce or eliminate hazardous substances. In the pharmaceutical sphere, this would likely mean APIs that are inactivated shortly or immediately after disposal or use. As the CE framework is being implemented in the EU, results in terms of both costs and outcomes would likely be revealed over the next several years. In the US, the EPA could play a lead role in addressing the patchwork of regulations and legislations that exist across different states governing disposal practices, with a consistent nationwide policy addressing environmental health and public safety [70] with extended partnerships and collaborations with industry groups such as the Pharmaceutical Research and Manufacturers of America, Cosmetic, Toiletry, and Fragrance Association, etc. and pave the way for strategizing and implementing a green pharmacy initiative.

## 6. The Future of Pharmaceutical Pollution and Environmental Justice

The growing creation of healthcare waste continues to contribute to increasing environmental pollution and its subsequent impact on human and animal health. There is a need to carefully examine environmental health disparities and how systems and policies create and perpetuate inequalities in exposure to environmental pollutants among vulnerable populations, thereby putting them at increased risk for disease and mortality. PPCP waste management is a specialized field that needs skilled and experienced staff to manage. A set of legislative guidelines that limit PPCP waste from manufacturing to usage to disposal should be endorsed by various regions with corresponding actions to reduce environmental contamination from PPCPs. The United Nations Sustainable Development Goals (UN-SDG) aim to protect the planet and ensure peace and prosperity for all by 2030. In order to achieve this, some of the first steps involve rethinking and re-examining waste. As we have discussed earlier, inequities in waste disposal impact the world’s most vulnerable populations. It is therefore critical to develop stronger policies and measures with specific and measurable targets to minimize environmental contamination and thereby exposure to pharmaceuticals.

## 7. From Pollution to Clean Ecosystems

‘Green pharmacy’ is the development of new substances that are more efficiently biodegraded but retain their effective pharmaceutical properties. The potential environmental impacts from the lifecycle of pharmaceuticals are not well-known and the trade-offs between biodegradability and medical effect should be agreed upon by various stakeholders. In looking at policies in three Western regions of the world, we find that there is, in general, a broader understanding and agreement on the impacts of pharmaceutical pollution, and efforts to mitigate risks have remained focused on wastewater treatment and disposal mechanisms along with reduced consumption through stewardship programs. Such efforts only address the demand side of pharmaceutical pollution. So far, only the EU has addressed the problem from the supply side by asking pharmaceutical manufacturers to provide Environmental Risk Assessments (ERA). However, it is difficult to estimate how many ERAs for human pharmaceuticals were conducted since the guideline was implemented in 2016. There is no publicly available record of ERAs and the responsibility for evaluation is divided between EMA and national competent authorities in Europe. During 2011–2012, the EMA administered and evaluated 42 ERAs, of which 20 required Phase II assessments [71]. It is clear that in order to reduce pharmaceutical pollution, cooperation will be needed across various sectors—manufacturers, regulators, healthcare services, etc., along with data that are shared publicly and is updated regularly.

Importantly, guidelines that address the entire lifecycle of pharmaceutical products need to be developed and enforced. The CE framework allows for innovations to enter at any step of the process. Thus, innovative products that are highly biodegradable or pipelines that can be made sustainable could easily become incorporated to develop better practices in reducing environmental pollution.

## 8. Conclusions

There is an urgent need for enhanced cooperation and collaboration amongst various agencies across nations, and in particular the US, to regulate pharmaceutical waste, including regulating the entire manufacturing cycle to reduce waste at the point of production. Current regulations and measures are falling short of reducing pharmaceutical contaminants in the environment. This may be partly due to a lack of reliable and relevant prospective risk assessment procedures as well as setting acceptable limits for APIs in the environment. Firstly, the amount of an ingested pharmaceutical that leaves the human body unaltered or in metabolized form is not well understood. Secondly, estimates of the relative contribution of excreted pharmaceuticals versus waste pharmaceuticals vary greatly, and, thirdly, ecotoxicology data are severely lacking for APIs and mixtures of APIs. Of the three regions mentioned in this paper, the EU is further along in addressing pharmaceutical disposal, API concentrations, and conducting risk assessments, yet much work needs to be done to uniformly measure and apply standards across all EU member states.

Likewise, particularly in the US and Canada, policies that address the entire life cycle of pharmaceutical products and mitigate environmental risk are critically needed, along with accompanying regulations and subsequent enforcement to mitigate environmental pollution. Broadly, efforts in this direction have included addressing the use and misuse of pharmaceuticals through programs such as antimicrobial stewardship, addressing excessive pharmaceuticals in households through take-back programs, and educating consumers regarding pharmaceutical waste. While each of these efforts may individually reduce waste to some degree, the problem of appropriate disposal and environmental consequences persists. Therefore, policies that encompass reductions in production, manufacturing, consumption, usage, and optimize waste disposal mechanisms are critically needed along with accompanying regulations and enforcements.

Several high and middle-income nations in Asia, Europe, Africa, and South America have developed policies and regulations governing healthcare waste that were beyond the scope of this paper. We end on an optimistic note regarding the growing awareness about green pharmacy and sustainable practices that may pave the way for rapid innovations. Future directions include a comparative analysis of policies specific to pharmaceutical waste handling and disposal across multiple nations for a global understanding.

## Figures and Tables

**Table 1:** Types of pharmaceutical waste.

Type	Description
Over-the-counter waste	Medications purchased over-the-counter without a prescription. (e.g., medications for colds, coughs, headaches, etc.)
Non-hazardous drug waste	Non-hazardous or non-controlled prescription medications. (e.g., diabetes, blood pressure medications, etc.)
Hazardous drug waste	Involves any waste that can potentially result in death or serious illness or pose significant hazards to human health or the environment if improperly stored, disposed of, transported, or treated. (e.g., chemotherapeutic agents). Hazardous drugs are highly regulated and must be collected and disposed of properly.
Controlled drug waste	Classified as drugs that are highly addictive and if taken in large amounts, can be toxic. (e.g., narcotics). Highly regulated and require disposal by a regulatory agency or company.
Veterinary and Agricultural use pharmaceuticals	Drugs used for veterinary and agricultural purposes. (e.g., antibiotics)

**Table 2:** Policies across the US, Canada, and Europe on pharmaceutical waste disposal.

	Manufacturing	Collection & Management	Tracking	Disposal	Environmental Concerns
**United States**	Toxic Substances Control Act (1976) Food and Drug Administration: Current Good Manufacturing Practices Federal Food, Drug, and Cosmetic Act (1938)	10-Step Blueprint for Healthcare Facilities (2019) Toxic Substances Control Act (1976)	Toxic Substances Control Act (1976)	EPA: Final Rule (2019)	Pharmaceuticals are being found in surface, ground, and drinking water around the country; there is rising concern about the possible adverse environmental consequences of these pollutants.
**Canada**	Environmental Impact Initiative (2001) Good Manufacturing Practices Priority Review of Drug Submissions Policy (2007)	Canadian Environmental Protection Act (1999) Food and Drug Regulations (Environmental Risk Assessment and Management of Ingredients in Drugs) (2022)	Environmental Impact Initiative (2001)	The Health Product Stewardship Association has return programs to facilitate the safe disposal of unwanted and used pharmaceutical products.	Pharmaceuticals have been found in soil and water. Their concentrations are low, however, they may negatively impact human and environmental health.
**Europe**	European Medicines Agency (EMA): ICH Q8 (R2) Pharmaceutical development (2009)	Directive 2004/27/EC	Article 8c of Directive 2008/105/EC EMA: Policy 78- Environment-al Policy	European Union Strategic Approach to Pharmaceuticals in the Environment (2019)	Remains of several pharmaceuticals have been found in surface and groundwaters, soils, and animal tissues across the Union.

**Table 3:** Pharmaceutical Pollution Policy Checklist.

***European Union***			
	**Yes**	**No**	**Maybe**
Are these countries/regions compliant with these policies currently?	X		
Are these laws or policies updated frequently? If yes, then explain how frequently.			X
Have these regional policies proven to have effective results? If yes, provide examples/citations.			X
Does the country have enacted laws or regulations around pharmaceutical waste?	X		
Is there an agency actively regulating these laws or policies?	X		
Are consumer perspectives on these policies considered?		X	
***Canada***			
	**Yes**	**No**	**Maybe**
Are these countries/regions compliant with these policies currently?	X		
Are these laws or policies updated frequently? If yes, then explain how frequently.			X
Have these regional policies proven to have effective results? If yes, provide examples/citations.			X
Does the country have enacted laws or regulations around pharmaceutical waste?	X		
Is there an agency actively regulating these laws or policies?	X		
Are consumer perspectives on these policies considered?			X
***United States***			
	**Yes**	**No**	**Maybe**
Are these countries/regions compliant with these policies currently?	X		
Are these laws or policies updated frequently? If yes, then explain how frequently.			X
Have these regional policies proven to have effective results? If yes, provide examples/citations.			X
Does the country have enacted laws or regulations around pharmaceutical waste?	X		
Is there an agency actively regulating these laws or policies?	X		
Are consumer perspectives on these policies considered?			X

## Data Availability

Not applicable.

## List of Abbreviations

AMR	Antimicrobial resistance
API	active pharmaceutical ingredient
CE	Circular Economy
CIPARS	Canadian Integrated Program for Antimicrobial Resistance
EC	European Commission
EU	European Union
EMA	European Medicines Agency
EPA	Environmental Protection Agency
PEC	predicted environmental concentration
PPCPs	pharmaceutical and personal care products
SDG	Sustainable Development Goals
EPPPs	environmentally persistent pharmaceutical pollutants
DEA	Drug Enforcement Agency
DOT	Department of Transportation
FDA	Food and Drug Administration
FWS	Fish and Wildlife Services
JC	Joint Commission
NOAA	National Oceanic and Atmospheric Administration
OSHA	Occupational Safety and Health Administration
SSRIs	selective serotonin reuptake inhibitors
US	United States
WHO	World Health Organization
